# The effect of daily consumption of different doses of fortified Lavash bread versus plain bread on serum vitamin-D status, body composition, metabolic and inflammatory biomarkers, and gut microbiota in apparently healthy adult: study protocol of a randomized clinical trial

**DOI:** 10.1186/s13063-019-3852-z

**Published:** 2019-12-27

**Authors:** Hadith Tangestani, Kurosh Djafarian, Sakineh Shab-Bidar

**Affiliations:** 1 Department of Community Nutrition, School of Nutritional Sciences and Dietetics, Tehran University of Medical Sciences (TUMS), No 44, Hojjat-dost Alley, Naderi St., Keshavarz Blvd, Tehran, Iran; 2 Department of Clinical Nutrition, School of Nutritional Sciences and Dietetics, Tehran University of Medical Sciences (TUMS), Tehran, Iran

**Keywords:** Vitamin D, 25(OH) D, Fortified bread, Fortification, Vitamin-D deficiency, Fortified food

## Abstract

**Background:**

Due to the high prevalence of vitamin-D deficiency worldwide and its health consequences, intervention studies at the community level are warranted. The present study has been conducted to evaluate the effectiveness of vitamin-D-fortified bread on serum vitamin-D levels, inflammatory and metabolic biomarkers, and gut microbiota composition in vitamin-D-deficient individuals.

**Methods/design:**

A double-blind, randomized controlled clinical trial is conducted on apparently healthy individuals with vitamin-D deficiency. The random allocation is done to divide participants into intervention groups including daily intake of vitamin-D-3-fortified bread (FB) with 500 IU/100 g bread (*n* = 30), FB with 1000 IU/100 g bread (*n* = 30), and 100 g plain bread (PD) (*n* = 30). At baseline and after 3 months of the intervention period, blood, stool, and urine samples are taken. Anthropometric measures, body composition, blood pressure, and dietary assessment are made. The gut microbiome composition is measured by the 16S rRNA approach. Data is analyzed by SPSS software version 21.

**Discussion:**

This study may partly explain for the first time the conflicting results from recent critical and systematic reviews regarding the role of vitamin D in microbiota composition.

**Trial registration:**

Iranian Registry of Clinical Trials (IRCT), ID: IRCT20170812035642N3. Registered on 11 March 2018; http://www.irct.ir/user/trial/28134/view.

## Background

Vitamin-D deficiency is one of the most important public health concerns worldwide. According to epidemiological maps in many parts of the world, about half of the population has insufficient serum levels of vitamin D due to insufficient exposure to sunlight and lack of dietary intake [1]. According to recently published data from Iranian National Food and Nutrition Surveillance, over 90% of the Iranian population have an undesirable serum level of vitamin D [2]. Vitamin D was taken into consideration because of its role in calcium-phosphorous and bone metabolism [3]. Thereafter, it became clear that inadequate vitamin D is associated with numerous pathological conditions such as autoimmune diseases like multiple sclerosis [4], type 1 diabetes [5], cancer [6], and even infectious diseases [7, 8]. The role of vitamin D in many tissues is known by its specific receptor, vitamin-D receptor (VDR). For example, the presence of VDR in immune system cells reflects the role of vitamin D in intestinal homeostasis and immunity [9].

The human gut is host for more than 10^14^ microorganisms, which are known as intestinal microbiota. These microorganisms play an effective role in obtaining nutrients, producing vitamins and destroying toxins by coexistence with the host [10, 11]. The intestinal microbiota is also effective in controlling pathogens [12], regulating intestinal immunity [13], and host metabolism [14, 15]. Based on previous studies, some of the factors affecting microbiome colonization, includes diet [16–18], host genetics [19], the environment [20], and medications such as antibiotics and laxatives [21].

Recently, a hypothesis has been suggested that vitamin-D deficiency can alter the composition of intestinal bacteria. A recently published observational study has shown a link between dietary intake of vitamin D and intestinal bacteria [22]. In mouse models, it has also been observed that vitamin D can alter the intestinal microbiota population into a beneficial bacterium [23]. It also seems that vitamin D plays a role in regulating the immune system in the gut, and its deficiency may change the function of the intestinal wall, leading to transfer of endotoxins, like lipopolysaccharides (LPSs), into the bloodstream, which itself causes the development of inflammation [24]. Animal studies have also shown that vitamin D reduces dysbiosis, infection, permeability of the intestinal epithelial duct and ultimately deacreases the incidence of inflammatory bowel diseases, like Crohn’s disease, by maintaining the hemostasis of the intestinal epithelium cells and tight-junction structures [23, 25, 26].

Vitamin D level is largely dependent on exposure to ultraviolet sun rays (wave-length of 290–315 nm). However, various environmental, cultural, social, and individual factors can affect the amount of ultraviolet B (UVB) that reaches the skin. On the other hand, dietary sources of vitamin D are too rare. Therefore, supplementation and fortification are the only ways to achieve the amount of vitamin D required. Supplement consumption is challenged because of population coverage, people’s compliance, cost, and regular consumption of supplements [27]. In contrast, using fortified foodstuffs has the advantage of lower cost and higher compliance [28]. Then, vitamin-D fortification seems to be a better startegy to prevent or treat vitamin-D deficiency. In this study we will fortify bread with vitamin D, because it is available to people with moderate to severe food-insecurity households and its use is not significantly related to socioeconomic factors such as income and education. Also, according to the latest survey of the national food pattern in Iran, the average consumption of bread is more than 300 g per day,while milk intake,another current food that is fortified, is only 38 g per day [29]. In addition, bread is a staple food which is consumed routinely in all meals by the Iranian population.

Considering the high prevalence of vitamin-D deficiency in Iran [30], and its contribution to chronic diseases [31], using a fortified food, such as vitamin-D3-fortified bread, if proved to be effective in this study, can be encouraged as a preventive tool. Moreover, studies on the role of vitamin D in the human intestinal microbiota are rare. Therefore, this study has been designed to test the effectiveness of vitamin-D-fortified bread on serum vitamin-D levels, inflammatory and metabolic biomarkers, and gut microbiota composition in vitamin-D-deficient individuals.

### Specific aim

The main aim of this study is to determine the effect of daily consumption of vitamin-D3-fortified bread on serum vitamin-D status, body composition, metabolic and inflammatory biomarkers, and gut microbiota in apparently healthy adults.

#### Primary objective

To compare within- and between-group variations in circulating 25(OH) D levels.

#### Secondary objectives

To compare within- and between-group variations in anthropometric, metabolic, inflammatory and oxidative stress biomarkers and gut microbiota

#### Hypotheses

1. Daily consumption of vitamin- D3-fortified -bread improves serum 25(OH) D levels in apparently healthy subjects.
2. Daily consumption of vitamin- D3-fortified -bread improves the biomarkers of anthropometric, metabolic, inflammatory, and oxidative stress and gut microbiota in apparently healthy subjects.

### Methods/design

#### Design

**Fig. 1:**
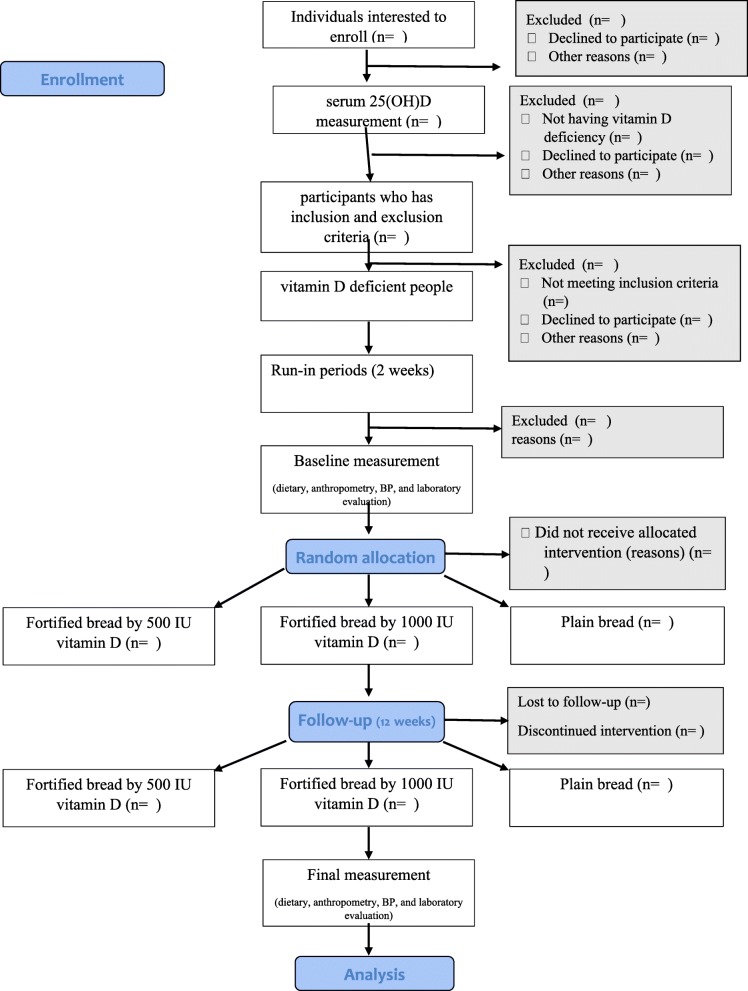
CONSORT flow diagram

This is a 3-month, double-blind, randomized controlled clinical trial (RCT) in which apparently healthy subjects aged 20–60 years are enrolled (Fig. 1).

#### Ethics, consent, and permissions

Tehran University of Medical Sciences will provide financial support for the current project. The trial will be conducted in compliance with the Declaration of Helsinki. The trial has received ethical approval from the Ethics Committee of Tehran University of Medical Sciences (number 96–02–161-35097). The current protocol is version 1, dated 31 January 2017, and any changes to the protocol will be communicated to all relevant parties, including participants.

#### Participant recruitment

In this trial, we will recruit a total of 90 participants (each group 30 subjects) from apparently healthy subjects of Tehran, the capital city of Iran. Participant recruitment was expected to commence by September 2018, and the trial is due to last 20 months. This assumes 4 months for participants recruitment, 4 months for run-in periods and intervention, 2 months for laboratory testing, 2 months for data collection, preparing and entering into SPSS software, 3 months for data retrieval and analysis, and 4 months to write the study report. A Standard Protocol Items: Recommendations for Interventional Trials (SPIRIT) Checklist [32] was used to report this protocol (Additional file 1, SPIRIT Checklist). Table 1 also shows the trial schedule. Before collecting any information, informed consent will be obtained from all participants. After reviewing the consent forms and answering any probable questions by study staff, those who are interested will be asked to sign the forms. A copy of the consent form will be gave to participants and all original signed consent forms will be kept by the study staff (Additional file 2, consent form).

**Table 1:**
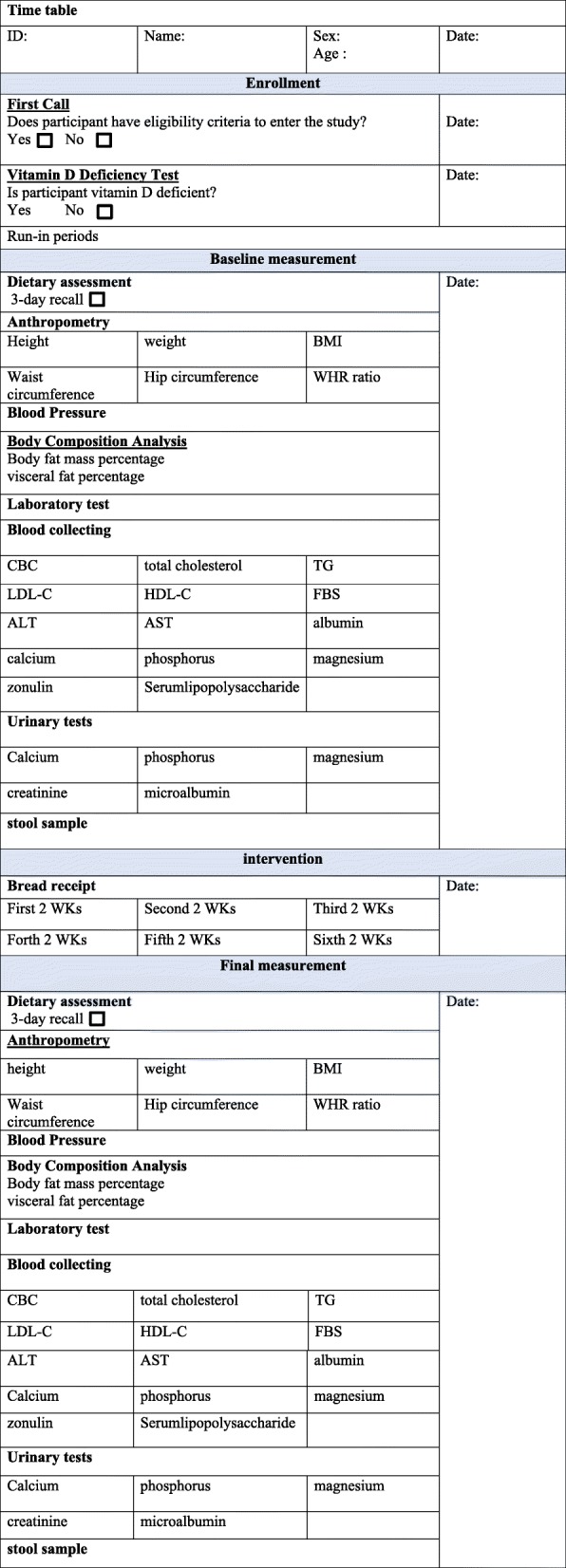
Timetable of protocol

*ALT* alanine aminotransferase, *AST* aspartate aminotransferase, *BMI* body mass index, *CBC* complete blood cell count*, FBS* fasting blood sugar, *HDL-C* high-density lipoprotein cholesterol, *LDL-C* low-density lipoprotein cholesterol, *TG* triglycerides, *WHR* waist to hip ratio

#### Eligibility

The inclusion criteria for subjects include (1) having vitamin-D deficiency (< 50 nmol/L), (2) no intake of supplements containing vitamin D or fish oil during the last 3 months, (3) not having clinical signs of diseases that interfere with vitamin-D metabolism (liver and kidney disease), and (4) not receiving any medications that interfer with the metabolism of vitamin D (corticosteroids, estrogen). Exclusion criteria include (1) any disease, or the need to use medication, which interferes with vitamin-D metabolism, (2) pregnancy or breastfeeding, and (3) any weight-loss treatments. Research participants can leave the study whenever they want.

#### Setting

The subjects will be recruited from the community by advertisements and notices, advertisements posted in public places, etc. For those who are interested in participating in the trial, study staff will confirm potential interest and eligibility of volunteers. Then, they will be reffered to a laboratory to identify subjects with vitamin-D deficiency. If they have the requirement criteria and express an interest to collaborate in the study, the appointment will be set for their first visit. At the first visit, the potential participants will have the proposals of the research project fully explained to them, and all interested participants will be asked to fill in the consent form. A general information questionnaire, including demographic information, disease history, type and amount of medicine used, occupation, smoking status, a sun-light exposure questionnaire, Physical Activity Questionnaire (PAQ), and a food recall questionnaire will be completed for each participant.

#### Sample size

Based on data on serum 25(OH) D changes from Nikooyehet al [33]., to achieve 20 nmol/L difference in circulating 25(OH) D with a two-sided alpha of 0.05 and a beta of 0.2 after a 12-week intervention period, the target sample size will be 30 individuals per treatment group. We are not adjusting the primary outcome comparisons for the multiple pairwise comparison.

#### Run-in

Subjects with vitamin-D deficiency who are volunteering to participate in the study will be entered into the 2-week maintenance weight program after the above steps have been taken. Subjects will be asked not to change their lifestyle and keep researchers informed of any changes including diet, physical activity, and medicine use. At the end of the first visit, a pamphlet will be given to the individuals, containing the description of the study, the description of the food groups, a date for the next visit, and the contact number. The presence of this run-in period is needed for maintening their weight, as well as to test the participants’ motivation to continue the intervention.

#### Randomization/blinding

Those participants with vitamin- deficiency (< 50 nmol/L) will be randomly assigned to receive either vitamin-D-fortified breads or plain bread. Randomization will be done using a computer-based random-digit generator. The allocation will be done in a 1:1:1 ratio. The research team, all participants, the staff involved in the clinical center, and those assessing the outcomes and analyzing the data will be blinded to group assignment. Letter codes A, B, and C wil be used to identify the three groups, respectively. Breads will be packaged in identical color, size, and taste. Therefore, neither the participants nor the researchers, the interviewers or laboratory staff know which participants belong to which group,

#### Intervention

Breads will be either plain (PB; containing no vitamin D/100 g bread) or fortified with two different dosages of vitamin D3 (FB; containing 500 IU vitamin D3/100 g bread or containing 1000 IU vitamin D3/100 g bread). Participants will receive a package of bread for both lunch and dinner. The timeframe for the intervention will be 12 weeks. Consumption of 1000 IU/day vitamin D is believed to be safe and effective to increase circulating 25(OH) D levels [34]. Participants will be given packages of bread for 2 weeks. All subjects will be visited biweekly to both assess their compliance and provide them with bread packages for the next 2 weeks.

#### Compliance

Participants will be asked to mark a reminder note for the daily consumption of bread. They will be given a sheet of paper that includes the code, the next visit date, two columns entitled “Lunch and dinner meals,” and another column entitled “Date” to record their regular consumption of bread. Patients will beinstructed to attach the sheet on the refrigerator in order to remind them of their use of the bread. Volunteers will return the sheet every 2 weeks and they will be provided with a new, 2-week, bread package.

#### Patient and Public Involvement

There is no involvement of patients or public in this study.

### Outcome measurements

#### Dietary assessment

A 3-day 24-h dietary recall questionnaire will be used to evaluate vitamin D and calcium intake. It includesd two work days and one weekend. Then, the data from questionnaire will be analyzed using N4 software (NUTRITIONIST 4, First Data Bank, San Bruno, CA, USA).

#### Anthropometry measures

The height will be measured without shoes by a stadiometer (Seca, Germany) with a sensitivity of 0.1 cm and weight with a digital scale (808 Seca, Germany) while wearing light clothes (with no coat or raincoat) with a sensitivity of 0.1 kg. Body mass index (BMI) will be calculated by dividing the weight (in kilograms) by height squared (in meters). The waist circumference will be measured by a strip tape midway between the lowest rib and the iliac crest and during expiration. Then the waist to hip ratio (WHR) will be calculated for each person.

#### Blood pressure

Blood pressure (BP) will be measured twice for each person using a digital barometer (BC 08, Beurer, Germany) after at least 10–15 min of resting in a sitting position. Then, the average BP for each person will be calculated and recorded.

#### Body composition analysis

Body fat mass and visceral fat percentage will be measured by bioelectrical impedance analysis (Quadscan 4000 system, Bodystat, UK).

### Laboratory investigations

#### Blood collecting

A 20-mL blood sample will be taken in a fasting state between 7:30 and 9:00 and stored in an acid-washed tube without anticoagulant. Then, after being stored at room temperature for 30 min and following blood clot formation, centrifugation at a speed of 1500 g for 20 min will be carried out. After that, the serum will be collected into separate micro-tubes and stored in a freezer at − 80 °C freezer until laboratory analysis.

#### Blood cell count

A complete cell blood count (CBC) will be measured by a blood-cell counter (Mythic, Orphee, France).

#### Blood chemistry

Fasting blood sugar (FBS) and lipid profile (total cholesterol, low-density lipoprotein cholesterol (LDL-C), high-density lipoprotein cholesterol (HDL-C), and triglycerides (TG)) will be measured by enzymatic methods based on colorimetry using commercial kits (Pars Test, Iran) using the automatic device (Selecta E, Vitalab, The Netherlands). Alanine aminotransferase (ALT) and aspartate aminotransferase (AST) will be also be measured in the serum by enzymatic assays. Measurement of serum concentrations of albumin, calcium, phosphorus, and magnesiumwill be performed based on colorimetric method, using commercial kits (Pars Test, Iran) and an automatic device (Selecta E, Vitalab, The Netherland). These tests will be performed for all patients on the same day of blood sampling.

The serum level of 25(OH) D will be measured by high-performance liquid chromatography (HPLC). Serum intact parthyroid hormone (iPTH) level will be measured using commercial kits DRG (Frauenbergstrasse 18 D-35039 Marburg, Germany) and using the enzyme-linked immunosorbent assay (ELISA) method.

#### Urinary tests

Calcium, phosphorus, magnesium, and creatinine will be measured using the colorimetric method, and microalbumin by the immunoturbidometric method using commercial kits (Pars Test, Iran) and automatic devices (Selecta E, Vitalab, The Netherland).

#### PBMC culture

Peripheral blood mononuclear cells (PBMCs) were separated using Ficoll-hypaque gradient density centrifugation and cultured as described elsewhere [30], with minor modifications. In this study, ~ 106 cells will be transferred to a culture well in a six-well cell-culture plate (Greiner bio-one, Germany), each well containing 1% phytohemmaglutinin (PHA) (Gibco), 100 ng/mL LPSs (Gibco, United States), 100 U/mL penicillin G and 10 ng/mL streptomycin (both from Sigma-Aldrich, United States) in a total volume of 2 mL RPMI 1640. PBMC culture is performed in triplicate.

#### Cytokine assay

Measuring the cytokines (serum highly sensitive C-reactive protein (hs-CRP) concentration, interleukin 4 (IL-4), interleukin 6 (IL-6), interleukin 10 (IL-10), tumor necrosis factor-α (TNF-α), and interferon gamma (INF-γ)) will becarried out in the culture medium.

#### Microbiota composition

The stool sample will be collected at baseline and at the end of the intervention and 16S ribosomal RNA (*16S rRNA*) genes, sequenced using polymerase chain reaction (PCR), will be applied to determine intestinal microbiome genome.

#### Gut permeability evaluation

Serum zonulin will be measured by ELISA. Serum LPSs will be measured using the HEK-Blue LPS Detection Kit (Invivogen, San Diego, CA, USA).

#### Safety procedures

We will record and report all adverse events, and participants with abnormal research samples will be referred to a physician.

### Data collection and management

Data from questionnaires will be collected by HT and two other trained questioners. The collected data will be reviewed by HT at the end of the day and, in the case of any discrepancies in data collection, the questioner will be mentioned to reduce bias.

### Statistical analyses

Data analysis will be done using SPSS version 21 software. To describe the quantitative data, the mean and standard deviations will be used and to describe the qualitative data absolute and relative abundance will be used. To compare absolute or relative abundance the chi-square or Fisher’s exact statistics will be used. As the data should compare groups, between groups, and between-group changes, and to reduce the probability of the error, an analysis of two-way variance with repeated measures will be used to explore the impact of time and time-group effects with adjustment using the Bonferroni post-hoc test. The effects of the intervention in each group were examined using the paired *t* test versus the baseline values. Between-group comparisons were examined using analysis of variance (ANOVA) followed by a Bonferroni post-hoc analysis. Covariance analysis will be used to adjust the effects of confounding variables. The correlation between the three groups will be evaluated by Pearson (normal data distribution) or Spearman (abnormal distribution). The significance level in this study will be considered to be *p* < 0.05.

### Discussion

In the last decade, attention has widened to the intestinal microbiota as an overlooked system. The various roles of vitamin D in health and many of its receptors in every organ of the body, particularly the gut, has increased the probable role of the intestinal microbiota through vitamin-D-dependent pathways in health and disease. Therefore, vitamin-D deficiency seems to play a vital role in health due to its effect on this intestinal microbiota. Solving the vitamin-D-deficiency problem through fortification of foods is an efficient and economical solution. Fortification has long been considered as an optional and mandatory way to improve nutrient deficiencies in developed countries. Bread has a special place in the Iranian diet. It is a staple food eaten with every meal and is also economically accessible to all people in the community. Moreover, it is important to note that milk, which is frequently fortified in developed countries, is not a good carrier for fortification in Iran due to its low consumption by the Iranian people. This study was designed following two important goals; the first is the efficiency of vitamin-D-fortified bread in improving serum levels of vitamin D and the second one is to determine the effect of vitamin D on body composition, metabolic and inflammatory biomarkers, and gut microbiota in apparently healthy adults.

The current protocol has some strength including the randomized placebo-controlled clinical trial design that is the strongest empirical evidence of a treatment’s efficacy and suitable for causal inferences. The second strength is the fortification of bread in two doses, which can more accurately reflect the treatment effects. Determining the role of vitamin D and intestinal microbiota also takes place for the first time in this study and can be considered as the third strongest point of this protocol. One of the limitations of this protocol is the inability to measure the total microbiota profile of the intestine due to lack of funding. In addition, our study is a short-term study and requires more pilot studies to be carried out for community-based fortification. Moreover, our protocol is based on apparently healthy people and cannot be generalized to the patient’s community.

### Trial status

The current protocol is version 1, dated 31 January 2017. The recruitment process will start on 1 July 2018 and is estimated to last for 6 months.

## Supplementary information

**Additional file 1.** SPIRIT Checklist.

**Additional file 2.** Consent form.

## Data Availability

The datasets generated and/or analyzed during the current study are available from the corresponding author on reasonable request.

## Abbreviations

ALT: Alanine aminotransferase
AST: Aspartate aminotransferase
BP: Blood pressure
BMI: Body mass index
CBC: Complete blood cell count
EDTA: Ethylene diamine tetra-acetate
ELISA: Enzyme-linked immunosorbent assay
FB: Vitamin-D-3-fortified bread
FBS: Fasting blood sugar
HDL-C: High-density lipoprotein cholesterol
HPLC: High-performance liquid chromatography
hs-CRP: Highly sensitive C-reactive protein
iPTH: Intact parathyroid hormone
IFN-gamma: Interferon-gamma
IL-4/IL-6/IL-10: Interleukin 4/interleukin 6/interleukin 10
LPSs: Lipopolysaccharides
LDL-C: Low-density lipoprotein cholesterol
N4: Nutritionist
PAQ: Physical Activity Questionnaire
PB: Plain bread
PBMC: Peripheral blood mononuclear cells
PCR: Polymerase chain reaction
PHA: Phytohemmaglutinin
RCT: Randomized controlled clinical trial
SPIRIT: Standard Protocol Items: Recommendations for Interventional Trials
TUMS: Tehran University of Medical Sciences
TG: Triglycerides
TNF: Tumor necrosis factor
UVB: Ultraviolet B
VDR: Vitamin-D receptor
WC: Waist circumference
WHR: Waist to hip ratio
16S rRNA: 16S ribosomal RNA
