# Supplementary material for: The effect of daily consumption of different doses of fortified Lavash bread versus plain bread on serum vitamin-D status, body composition, metabolic and inflammatory biomarkers, and gut microbiota in apparently healthy adult: study protocol of a randomized clinical trial
Source: Trials. 2019 Dec 27;20:776. doi: 10.1186/s13063-019-3852-z (PMC6935154; doi:10.1186/s13063-019-3852-z)
Supplement: Supplementary file 2 — Additional file 2. Consent form. [file 13063_2019_3852_MOESM2_ESM.docx]

**
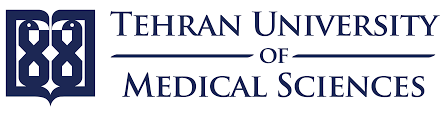
**

**Informed consent**

Mr / Ms….
We invite you to participate in our research. Information about this research is provided in this sheet and you are free to participate in this research.

You do not have to make an immediate decision. You can ask your questions from the research team to make a good decision, and can consult with anyone you want. Before signing this consent, make sure you understand all the information in this form and answer all your questions.

**Research title:** The effect of daily consumption of different doses of fortified Lavash bread versus plain bread on serum vitamin D status, body composition, metabolic and inflammatory biomarkers, and gut microbiota in apparently healthy adults

- I know that the main aim of this study is to identify the effect of daily consumption of vitamin D fortified bread on serum vitamin D status, body composition, metabolic and inflammatory biomarkers, and gut microbiota in apparently healthy adults
- I know my company is completely voluntary in this research and I do not have to participate in this research. I was assured that if I was not willing to participate in this research, I would not be deprived of routine diagnostic and therapeutic care and my connection with the center and physicians do not get bored.
- I know that even after agreeing to participate in the research, I can leave the research whenever I want, albeit after informing the researchers. Moreover, my abandoant from the research will not deprive me of the usual services for me.
- My cooperation in this research is as follows:

I know that if I have the inclusion criteria of the research (age 20-60 years, lack of vitamin D supplementation or fish oil in the last three months, lack of any diseases like kidney and liver disease, lack of medication especially corticosteroid medications, and Estrogen, and pregnant and lactating), I can participate in this study so I respect honesty in answering questions. Then, to determine the status of the serum vitamin D, I must go to the laboratory that the researchers determine. If I get vitamin D deficiency, I can take part in this research, and if I do not have vitamin D deficiency, I cannot continue this research. Then, for two weeks, I have to follow the researchers recommendations, including avoiding any weight loss diets and severe physical activity. After 2 weeks I will undergo the research. Researchers will take me 10 ml of my blood pressure to test, blood glucose, serum lipid profile, albumin and urine creatinine, and etc. I need to be at least 10 hours fasting for blood sample. I will also be taken samples of urine and stool. Questionnaires on nutritional status and socioeconomic status, exposure to sunlight and physical activity will be completed for me. Anthropometric and blood pressure tests will also be performed. It at least takes me two hours to complete the questionnaires. I know that the study consists of three groups. People in group 1 will receive 100 grams of bread each day fortified by 500 international units of vitamin D, individuals in group 2 will receive 100 grams of breads per day fortified by 1000 units of international vitamin D and group 3 will receive 100 grams of plain bread per day. I randomly took one Three groups of research. My placement in each of the three groups will be completely randomized, and I cannot be involved in determining the research groups. I agree to randomly place in a bread groups. I know that the duration of this study is 3 months, during which time I will have to change 100 grams of daily bread consumption with the lavash bread the researchers will give me. All tests include blood, urine, and stool sample, anthropometric measurements, and blood pressure, as in the beginning of the study done, are repeated at the end of the study. So, I know that 10 ml of blood, which requires at least 10 hours of fasting will be taken (2 times) at the beginning and end of the research. Researchers pledge me to compensate my travel expenses to the clinic by free report of the test results.

- The potential benefits of my company in this study are as follows:
  The probable benefits of my company in this study is it reveal my vitamin D deficiency problem. Moreover, other diagnostic tests, such as blood glucose measurements, blood lipids, etc., will be done for me two times, which is reported to me free of charge. Also, the amount of 100 grams of daily bread intake for 3 months from this study can be provided, which can help to resolve my vitamin D deficiency. By participating in this project, I can also receive free advice and strategies for treating vitamin D deficiency after the end of the project. In addition, my partnership in this research can be helpful in identifying treatment options for vitamin D deficiency, which is a global problem
- The damages and possible complications of this study are as follows:
  The injuries and possible side effects of the company in this study are as follows: This study does not cause any harm to me and does not have any side effects. Also, the amount of blood sample taken does not hurt me. Involvement in this research not only does not hurt me, it can be useful in diagnosing and treating my vitamin D deficiency. By participating in this project, due to my diagnosis of vitamin D deficiency, my treatment may be postponed for 3 months, which will not cause me a big problem. And the administrators will assure me that, at the end of the research, I will provide the necessary treatment and dietary recommendations to solve this problem.
- If I do not want to participate in the research, I will provide the usual services (therapeutic, diagnostic, etc.), I will be presented with the usual method of treatment, and my routine treatment will continue.
- I know that the researchers of this study kept all my information confidential and are only allowed to publish the general results of this research without mentioning my name and profile.
- I know that the Ethics Committee of Research to monitor my rights can access my information.
- I know that this research costs me nothing.
  My company is totally voluntary in this study.
- I know that participation in this research does not cost me. By participating in this research, vitamin D deficiency tests (at the beginning and end of the study) as well as other tests, such as blood sugar measurement, blood lipids, Blood pressure, anthropometric measurements (height, weight, etc.) 2 times (at the beginning and end of the study) are done for me free of charge. Also, during the study, I often receive free nutrition assessment and advice. Also, all tests will be free and will not have any side effects.
- Dr. Sakineh Shab-Bidar and Hadith Tangestani are introduced to me to answer my questions and I can ask for guidance whenever a problem or question related to the participation in the research came about.
  The following address and telephone number were provided to me as follows:
  Address: No 44, Hojjat-dost Alley, Naderi St, Keshavarz Blvd, Tehran, Iran

Tele: 021-88955742
Mobile: 09111376516

- I know that if any physical and mental problems arose during and after the research because of my participation in this research, it will be the responsibility of the practitioner to treat its complications and the related damages.
- I know if I have a problem to participate or in the research process, I can contact the Ethics Committee of Tehran University of Medical Sciences at Tehran University of Medical Sciences at 605 Room, Qods Street, Keshavarz Blvd address.
- This information form and informed consent will be provided in two copies and after the signature, a copy will be available to me and another copy to the researchers.

**I read and understood the above, and based on that, I declare my informed consent to participate in this research.**Participant Sign

**Dr. Sakineh Shab-Bidar as an administer of this research is committed to do all the items mentioned above and provided the safety of all participants.**

Administer sign
